# Aging-Enhanced High-Performance Zinc Tin Oxide Transistors and Exploration in Illumination Interface Stability

**DOI:** 10.3390/nano16140861

**Published:** 2026-07-13

**Authors:** Bing Yang, Qiao Guo, Hongmin Li, Gang He, Shanshan Jiang, Longwei He, Xiang Li, Peng Yu

**Affiliations:** 1 School of Intelligent Manufacturing, Anhui University of Applied Technical, Hefei 230011, China; 2 School of Computer and Information Technology, Anhui University of Applied Technical, Hefei 230011, China; guoqiao@uta.edu.cn; 3 Hefei BOE Optoelectronics Technology Co., Ltd., Hefei 230011, China; 4 Radiation Detection Materials & Devices Lab, Anhui University, Hefei 230601, China; 5 Pioneer Film Materials (Anhui) Co., Ltd., Hefei 230011, China

**Keywords:** aging-enhanced thin-film transistors, grain boundary defects relaxation model, illumination interface stability

## Abstract

In this work, a post-annealing rapid cooling and aging treatment process is innovatively proposed to build high-performance zinc tin oxide (ZTO) thin-film transistors. The relaxation effect on the abundant oversaturated oxygen vacancy deep-level traps contributes to the shallow donor formation during the aging period. The TFTs aged in an air environment for 10 days possess significantly improved electrical performance, including a clearly increased on/off current ratio of 7 × 10^6^ from 2 × 10^4^ and a markedly increased saturation mobility of 5.9 from 2.2 cm^2^·V^−1^·s^−1^, verifying the facile method to improve the electrical property of polycrystalline TFTs, and the method has been investigated using the grain boundary defect relaxation model and energy band theory. It is worth mentioning that the TFTs aged under vacuum conditions realized more effective regulation and control on off-state current and demonstrated a wider aging time window. The distinctive illumination interface stability was studied in depth using a charge trapping model and electron–hole pair model, which embody the potential application in photoelectric detectors.

## 1. Introduction

Nowadays, researchers are keen on developing various emerging thin-film transistor (TFT) applications, including Virtual Reality/Augmented Reality transparent display [1], flexible wearable devices [2], pressure sensors [3], neuromorphic computing and nonvolatile memories [4,5]. With the reduction in the lateral size of TFTs, researchers have tried a variety of alternative dielectric materials to silicon dioxide to increase the gate capacitance and reduce the operating voltage via High-K Metal Gate (HKMG) technology [6,7,8,9,10,11,12,13,14]. As the mainstream channel material, In-based TFTs have become a research hotspot in recent decades due to the unique overlapping In atomic 5 s orbital, which allows for the expansion of the conduction band and the attainment of excellent electrical performance [10]. For example, Shan et al. successfully fabricated 600 °C-annealed In_2_O_3_/SrO_x_ TFTs, which exhibit a field-effect mobility of 5.61 cm^2^ V^−1^ S^−1^ at an operating voltage of 3 V [11]. Guo et al. reported that In_2_O_3_/Yb_2_O_3_ TFTs annealed at 500 °C possess a saturation mobility of 4.98 cm^2^ V^−1^ S^−1^ [12]. However, the exorbitant cost has limited In-based TFTs’ applications in large-scale industrial production. As a more abundant and non-toxic biodegradable material on Earth, the ZnSnO (ZTO) thin film has been considered to be a promising cost-effective replacement for In-based metal oxides for the reason that Sn^4+^ possesses a similar electron configuration of (*n* − 1) d^10^ *n*s^0^ with In atomic 5 s orbital, thus the ZTO thin film has been selected as the channel layer in this thesis [13]. Recently, a variety of dielectric materials, such as Yb_2_O_3_ [6,12], DyO_x_ [7,8], HfO_2,_ [10], SrO_x_ [11], Ga_2_O_3_ [14], Ce_2_O_3_ [15], and ZrO_2_ [16,17,18,19,20], and their mixtures [9,10,15,18] have been explored using HKMG technology. Compared with other high-k dielectric materials, ZrO_2_ and HfO_2_ films demonstrate wider band gaps and excellent stability, which can significantly improve the electrical performance and reliability of oxide TFTs, ultimately meeting the strict requirements of high-resolution and high-refresh-rate display panels for device performance. The latest research indicates that ZrO_2_ exhibits a smaller capacitance equivalent thickness (CET) and a higher dielectric constant in comparison to HfO_2_ on account of the formation of highly oriented t/c/o crystalline phases [17]. Therefore, ZrO_2_ films are chosen as dielectric layers of ZTO/ZrO_2_ TFTs in this study. Recently, Yang et al. successfully utilized the self-developed complicated atomic-layer deposition (ALD) system to prepare ZTO/ZrO_2_ TFTs and carbon nanotube (CNT) CNT/ZrO_2_ TFTs; corresponding high mobilities of 8.5 and 3.6 cm^2^ V^−1^ S^−1^ were achieved at a high operating voltage of 10 V. Based on this, they successfully constructed a complementary metal-oxide–semiconductor (CMOS) inverter [18]. Nevertheless, the exorbitant equipment costs and extremely slow deposition rate limit large-scale device production. Jang et al. developed high-performance flexible ZrO_2_/SnO_2_ TFTs with an on/off current ratio of 1.13 × 10^6^ at a low operating voltage of 3 V using combustion synthesis [19]. Although the reported combustion-assisted methods contribute to obtaining low-temperature fabricated ZrO_2_ TFTs, the extreme reaction makes it difficult to obtain precise process parameters due to the poor film formation uniformity and a large amount of oxygen vacancy defects [1]. Lee et al. introduced a new method for synthesizing sol–gel ZrO_2_ films through a ligand exchange strategy and successfully fabricated a fully oxide-patterned solution-processed InZnO TFT with a high mobility of 39.6 cm^2^ V^−1^ S^−1^ for the first time [20]. Compared with various preparation methods, spin-coating technology has been considered as the most facile method for TFT performance exploration and is chosen in this study. Previously reported ZnSnO solution-based TFTs usually demonstrate poor saturation mobility of ~2.5 cm^2^·V^−1^·s^−1^ [8,9], which has limited their applications on high-performance devices; thus, we will try various methods to improve the electrical performance. In addition, Long et al. systematically investigated the time-dependent dielectric breakdown reliability of ZrO_2_ dynamic random access memory capacitors [21]. However, a comparison of TFT reliability in vacuum and air environments has not yet been reported, which is of crucial importance for the device packaging process.

In this study, we report on aging-enhanced high-performance ZnSnO/ZrO_2_ TFTs that have been successfully fabricated and intensively studied in terms of aging stability and illumination stability. The deep-level traps resulting from the oversaturated oxygen vacancies lead to the carrier transmission being blocked during the post-annealing rapid cooling stage. The oversaturated traps will convert to shallow donors through defect relaxation as well as the H_2_O molecular donor role, which will benefit electronic quick transport. The 10-day air-ambient aged ZnSnO/ZrO_2_ TFT demonstrates excellent electrical properties, including a high on/off current ratio of 7.0 × 10^6^ and large saturation mobility of 5.9 cm^2^·V^−1^·s^−1^, which even surpasses some reported pricey In_2_O_3_/ZrO_2_ TFTs (3.08~4.42 cm^2^·V^−1^·s^−1^) [22,23]. However, the aged TFTs under vacuum conditions realized more effective regulation and control in off-state current, which displays a wider aging time window (20 days). The distinctive Δ*V*_TH_ extracted from various illumination experiments can be used for detecting diverse illuminating lights and demonstrate potential application in photoelectric detectors.

## 2. Materials and Methods

The 0.1 M ZrO_2_ solution was prepared by dissolving 322.25 mg ZrOCl_2_·8H_2_O (Aladdin™ Z164495 Zirconyl chloride octahydrate, Aladdin Scientific, Riverside, CA, USA) in 10 mL 2-methoxyethanol (2-ME, (Aladdin™ M684845 Ethylene glycol monomethyl ether). The 0.2 M ZTO precursor (Zn/Sn = 1) was obtained by mixing 219.5 mg Zn(CH_3_COO)_2_·2H_2_O (Aladdin™ Z433041 Zinc acetate dihydrate) and 225.7 mg SnCl_2_·2H_2_O (Aladdin™ T431118 Tin(II) chloride dihydrate) in 10 mL 2-ME All the chemical reagents were purchased from https://www.aladdin-e.com/. After 6 h stirring and 24 h aging, the transparent ZrO_2_ solution was spun twice on the heavily doped p-type Si wafers (Aladdin™ S1508675 Silicon Wafer) with 5000 rpm(revolutions per minute) after 120 s plasma cleaner. After baking at 180 °C for 10 min and undergoing UV (Ultraviolet Rays) treatment for 40 min, the as-deposited films underwent annealing treatment from 460 °C to 610 °C for 1 h. Then, the ZTO precursor was spun at 6500 rpm on the samples and annealed at 320 °C, 430 °C and 540 °C for 1 h then rapidly cooled to room temperature. The pure aluminum wires (5N purity grade, produced by Hebei Jiuyue Advanced Materials Technology Co., Ltd., Shijiazhuang, China) were patterned as the drain and source electrodes through thermal evaporation process. To avoid the mobility being overrated, the length (*L*) and width (*W*) of channel were defined as 100 and 1000 μm by the mask.

All containers must first undergo ultrasonic cleaning by SHUMEI KQ-100DE Numerical Control Ultrasonic Cleaner to eliminate experimental errors caused by impurities, which is made by Kunshan Ultrasonic Instruments Co., Ltd. (Kunshan, China). Except the conventional ultrasonic cleaning, the wafer was subjected to standard RCA cleaning. The plasma cleaner can be finished via Harrick PDC-002 series plasma cleaner manufactured by Harrick Plasma (Ithaca, NY, USA). The stirring process can be operated via HH-8J magnetic stirring water bath pot which is made by JSTLIANGYOU (Liyang, China). The spin-coating process can be operated via Spin Master-100 spin processor which is designed by Chemat Technology Inc. (Los Angeles, CA, USA), and the manufacture process has been shift to Shang Hai of China by Its subsidiary. The baked and anneal process can be engaged via the HOT PLATE (model K W-4AH) fabricated by CHEMAT TECHNOLOGY, INC. (Los Angeles, CA, USA). The UV treatment can be operated via AHD-1KW/330MMUV UV curing machine manufactured by Shenzhen Anhongda Optoelectronic Technology Co., Ltd. (Shenzhen, China). Weight loss, film thickness, crystallization process, morphology, and chemical bonding states of ZrO_2_ can be obtained via TGA5500 thermogravimetric analysis (TGA), SC-630 spectroscopy ellipsometry, SmartLab 9kW grazing incidence X-ray diffraction (GIXRD), Hitachi 5500M Atomic force microscopy (AFM) and ESCALAB 250Xi X-ray photoelectron spectroscopy (XPS). The TGA5500 is manufactured by TA Instruments (New Castle, DE, USA). The SC-630 spectroscopy ellipsometry is manufactured by Shanghai Sankun Instrument Co., Ltd., Yangzhou, China. The SmartLab 9kW GIXRD is manufactured by Rigaku Corporation located in Tokyo, Japan. The Hitachi 5500M AFM is manufactured by HITACHI High-Tech (Tokyo, Japan). The ESCALAB 250XiXPS is manufactured by Thermo Fisher Scientific (Waltham, MA, USA). The band gaps and transmittance of ZrO_2_ can be obtained via the same spin-coating process on the quartz substrates and explored by UV2550 ultraviolet–visible spectrophotometer (UV–vis) manufactured by Shimadzu Corporation (Kobe, Japan). The electrode thermal evaporation and vacuum aging samples preservation can be achieved via the PVD500 High vacuum magnetron sputtering thin film deposition System produced via the Shenyang Scientific Instruments Co., Ltd., Chinese Academy of Sciences located in Shenyang of China. The air aging samples preservation can be realized via preserving the samples in HBX-1000LHWHS components temperature and humidity-controlled storage cabinet with a relative humidity (RH) of 40% and a fixed temperature of 20 °C, which is fabricated by Shenzhen Huaboxu Technology Co., Ltd. (Shenzhen, China). The electrodes of integrated MOS capacitors and TFTs should be connected with the Cascade Microtech EPS 150 probe station fabricated by the Cascade Microtech (Beaverton, OR, USA). The electrical property can be acquired via Keithley E4990A, manufactured by Keysight Technologies (San Jose, CA, USA); Keithley 2636B, manufactured by Keithley Instruments, Inc. (Cleveland, OH, USA); and Agilent B1500A, manufactured by Keysight Technologies (San Jose, CA, USA). The illumination stability can be explored via the CME-Mo301 monochromator with the CME-X305F xenon lamp source for TFT photoelectric test, which is integrated by Zhongke Weineng (Beijing) Technology Co., Ltd. (Beijing, China).

## 3. Results and Discussion

### 3.1. Physical and Chemical Properties of ZrO_2_

**Figure 1:**
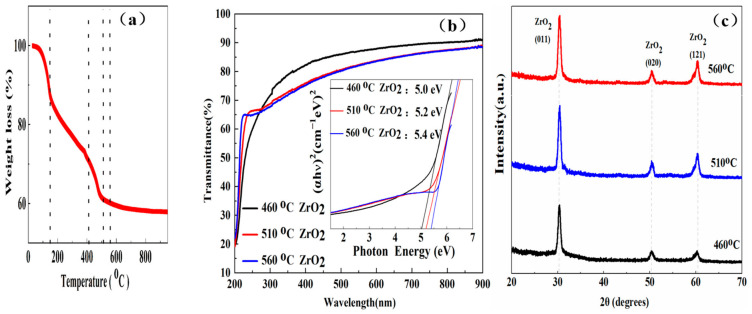
(**a**) The TG results of ZrO_2_ xerogel. (**b**) Optical transmittance change in ZrO_2_ thin films with the annealing temperature increasing from 460 to 560 °C. The inset demonstrates the Tauc plots and the fitting optical band gaps of the ZrO_2_ thin films. (**c**) XRD patterns of the ZrO_2_ as functions of annealing temperatures.

To probe the thermal decomposition properties of ZrO_2_ xerogel, thermogravimetric (TG) analysis was performed with a heating rate of 5 °C min^−1^, as shown in Figure 1a. The leftmost dashed line demonstrates that the first significant weight loss occurs at 150 °C, which can be ascribed to the residual solvent evaporation. The second obvious weight loss at 410 °C (the position of the second dashed line on the left) can be attributed to dihydroxylation. The third significant weight loss implies the formation of stable metal oxides begins at 510 °C (the position of the third dashed line on the left). There is no obvious decrease when the annealing temperature surpasses 560 °C (the position of the fourth dashed line on the left), which implies that the samples have been fully converted into ZrO_2_ metal oxide [9].

To explore the potential applications in transparent devices, the ZrO_2_ films were spun on the quartz substrates with various annealing temperatures ranging from 460 to 560 °C. The fitting optical band gaps and transmittance are displayed in Figure 1b. All samples demonstrate large transmittance exceeding 70% in the visible range of 390 nm–780 nm, indicating their possible applications in transparent devices. The optical band gap (Eg) continues to rise from 5.0 eV to 5.4 eV as the annealing temperature increases from 460 to 560 °C, which means that the electronics in the dielectric layer hardly transmit from the valence band to the conduction band, implying excellent leakage performance [7].

As demonstrated in Figure 1c, the 460 °C-annealed ZrO_2_ sample has obvious representative crystalline peaks corresponding to (011), (020), (121) lattice planes at diffraction angles of 30.22, 50.32 and 60.29, which match well with the JCPDS 17-0923 ZrO_2_ (tetragonal zirconia) International Centre for Diffraction Data (ICDD), Swarthmore, USA, 1997, demonstrating the representative crystallization procedure [23].

**Figure 2:**
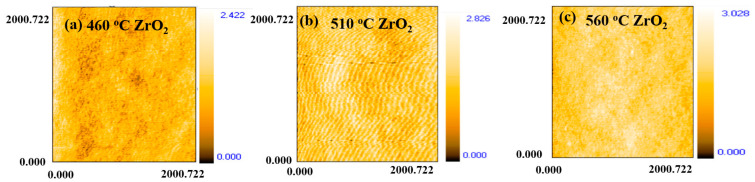
AFM images of ZrO_2_ thin films with the annealing temperature at (**a**) 460 °C, (**b**) 510 °C and (**c**) 560 °C.

To investigate the annealing temperature impacts on the morphology of ZrO_2_ thin films, samples with the same square and different heights were measured via AFM, as shown in Figure 2. When the annealing temperature increases from 460 to 560 °C, the root-mean-square (RMS) roughness of ZrO_2_ samples is 0.233, 0.221, 0.153. The 460 °C-annealed ZrO_2_ sample has a high RMS of 0.233 and implies a mass of nanopores has been generated from residual organic ligands. The 560 °C-annealed ZrO_2_ sample demonstrates a low RMS of 0.153 and means a smooth surface is available, contributing to the electronics quick transmission, which will lead to a leakage performance improvement [8]. 

**Figure 3:**
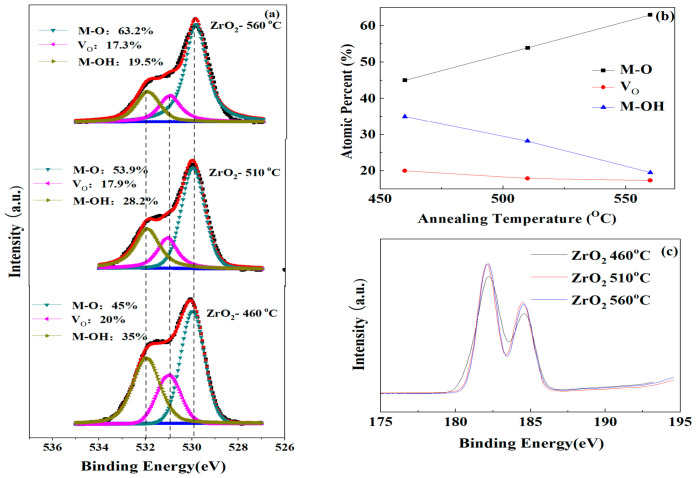
(**a**) XPS spectra of O1s peaks for ZrO_2_ thin films as a function of annealing temperature. The vertical dashed lines correspond to the fitted three peak positions. (**b**) Semiquantitative analyses of the oxygen component for the corresponding ZrO_2_ thin films with the annealing temperature increasing from 460 to 560 °C. (**c**) XPS spectra of Zr 3D peaks for ZrO_2_ thin films as a function of annealing temperature.

The XPS measurements were performed to detect the correlation between the chemical bonding states and annealing temperatures. As a reference, the C 1s peak located at 284.8 eV was used to calibrate all the experimental results. As demonstrated in Figure 3a, the O1s peaks of ZrO_2_ films can be fitted into three peaks located at 530, 531, and 531.96 eV, which are attributed to the oxygen in oxide lattices (M-O), oxygen vacancy (V_o_), and hydroxide (M-OH), respectively. As shown in Figure 3b, when the annealing temperatures increases from 346 to 560 °C, the fraction of M-O progressively increases from 45% to 63.2%, which implies that more electronic transmission paths are available. Meanwhile, the continuous decreasing fraction of M-OH as well as the slight reducing V_o_ can be noticed, which means that the lessening defects in the forbidden band and benefit to the electronic transport. Peak deviations of Zr 3d_3/2_ and Zr 3d_5/2_ toward lower binding energy can be noticed in Figure 3c, which can be attributed to the diffusing oxygen in films and the forming ZrO_2_ samples. Insufficient scanning times and the low signal-to-noise ratio of the original O1s spectrum led to the fitting algorithm misidentifying noise as real signals. Naturally, the fitting curve generated by the algorithm to match the noise cannot fully coincide with the original spectrum’s actual contour [9].

### 3.2. Electrical Performance Analysis of Integrated MOS Capacitors, TFTs and Inverters

**Figure 4:**
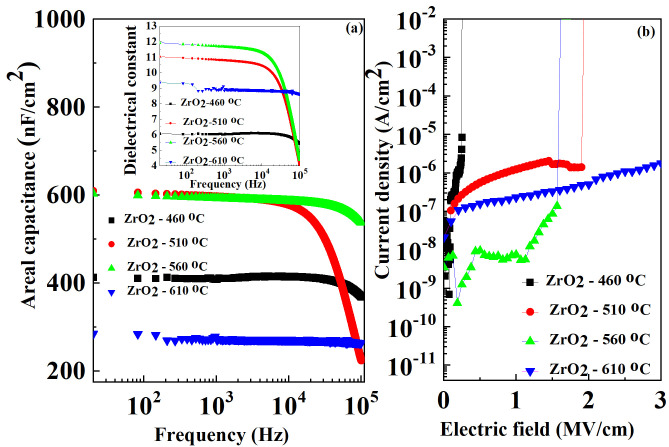
(**a**) Areal capacitance of the ZrO_2_ thin films with the annealing temperature increasing from 460 to 610 °C. The inset demonstrates the corresponding dielectric constant changes. (**b**) Leakage current density and breakdown electric field of the ZrO_2_ thin films with the annealing temperature increasing from 460 to 610 °C.

The Al/ZrO_2_/Si gate stack MOS capacitors have been integrated to investigate the dielectric and leakage properties. As shown in Figure 4a, with the annealing temperature increasing from 460 to 560 °C, the calculated areal capacitance rises from 415.76 to 600 nF/m^2^, which can contribute to the addition of dense metal compounds. However, when the annealing temperature increases to 610 °C, the areal capacitance sharply decreases to 285.69 nF/m^2^, caused by the formation of low-k metal silicates [23]. For high-resistivity oxides like ZrO_2_, the low-frequency impedance can reach the order of MΩ or even GΩ, which exceeds the current measurement range of the impedance analyzer. As a result, the phenomenon of “discontinuity” in the low-frequency region curve can be observed [9]. According to the fitting film thickness of 12.8, 15.7, 17.3 and 29 nm, the corresponding dielectric constant (k) was calculated to be 6.1, 11.0, 11.7 and 9.3, as shown in the inset of Figure 4a. With the increase in annealing temperature from 460 to 560 °C, the constant reducing leakage current density and continuously increasing breakdown in the electric field can be noticed, as displayed in Figure 4b. Nevertheless, the leakage current density increased from 5.1 × 10^−9^ to 2.8 × 10^−5^ A/cm^2^ when the annealing temperature rose from 560 to 610 °C, which verified the aforementioned low-k metal silicates. Hence, the 560 °C-annealed ZrO_2_ sample equipped with the most excellent leakage performance was selected as the dielectric layer. It is worth noting that the filtered water-induced ZrO_2_ film can also achieve similar dielectric properties. However, its poor film-forming performance is prone to generating the low breakdown electric field [9].

**Figure 5:**
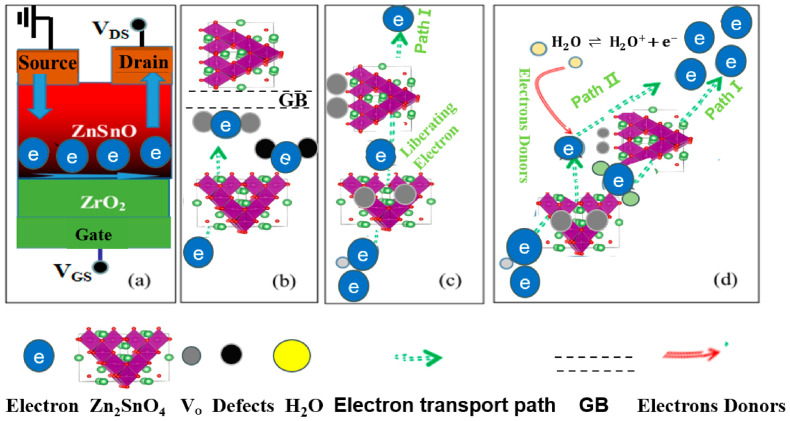
(**a**) Bottom-gate and top-contact architecture TFTs. (**b**) Trapped electronics in the grain boundary. (**c**) Liberating electronics attributed to the defects relaxation in vacuum environment. (**d**) Liberating electronics attributed to the oxygen vacancies relaxation and H_2_O molecular electrons donor role in air-ambient environment.

The ZTO films with different annealing temperatures were integrated with 560 °C-annealed ZrO_2_ films to fabricate bottom-gate top-contact ZrO_2_/ZTO TFTs, as illustrated in Figure 5a. The channel electrons were trapped in the grain boundary, as shown in Figure 5b. The liberating electronics realize quick transport after aging in vacuum and air-ambient environments, as shown in Figure 5c,d. The crucial electrical parameters, including the threshold voltage (V_TH_) and current on/off ratio (I_on/off_), can be obtained from transfer characteristics; the sub-threshold slope (SS), the saturation mobility (μ_sat_) and the dielectric constants (k) can be computed from the following equations [7]:

(1) $$\mu_{\mathrm{sat}}=\frac{2L}{WC_{i}}(\frac{\partial \sqrt{I_{DS}}}{\partial V_{GS}}{)}^{2}$$

(2) $$\mathrm{SS}=\frac{\partial V_{\mathrm{GS}}}{\partial (\ln I_{\mathrm{DS}})}$$

(3) $$C_{i}=\frac{\varepsilon_{0}kS}{d}$$

where C_i_ represents calculated areal capacitance. To obtain an accurate C_i_ value and ensure the accuracy of the μ_sat_, we prepared the MOS capacitors with the same batch and process conditions as the TFTs. The I_DS_ represents the current from drain to source electrodes; the V_GS_ represents the imposed voltage between gate and source electrode; the d represents the fitting thickness of annealed ZrO_2_ samples; the S is 0.0144 m^2^ on the basis of the masking template; ε_0_ represents the value of 8.85 × 10^−12^ F/m on account of the permittivity of free space [11]. The key parameters containing the μ_sat_, I_on/_I_off_, V_TH_ and SS are listed in the Table 1.

**Figure 6:**
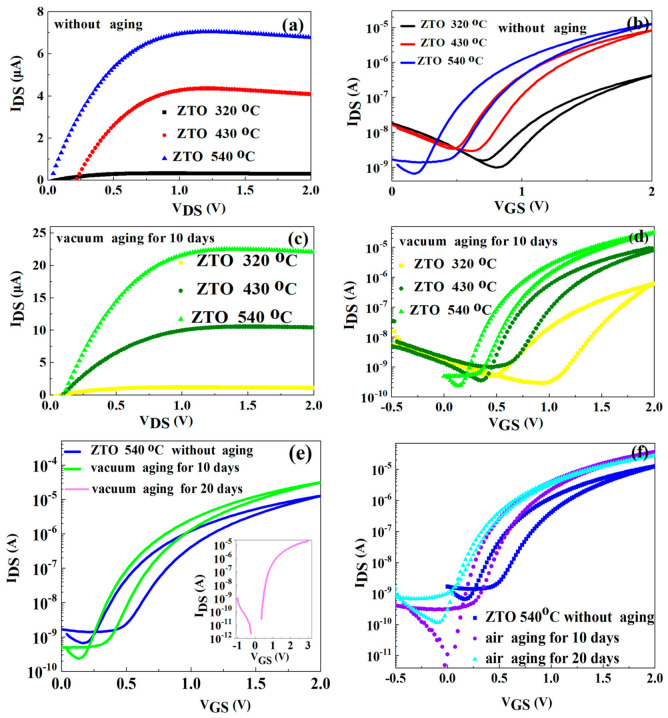
(**a**) Output characteristics and (**b**) transfer characteristics of the ZnSnO/ZrO_2_ TFTs as a function of annealing temperature of ZnSnO channel layer without aging. (**c**) Output characteristics and (**d**) transfer characteristics of the ZnSnO/ZrO_2_ TFT aging in vacuum environment for 10 days as a function of annealing temperature of ZnSnO channel layer. (**e**) Transfer characteristics comparison between the 540 °C-annealed ZnSnO/ZrO_2_ TFTs without aging and aging in vacuum environment for 10 days and 20 days (shown in the inset). (**f**) Transfer characteristics comparison between the 540 °C-annealed ZnSnO/ZrO_2_ TFTs without aging and aging in air environment for 10 days and 20 days.

Figure 6a,b reveal the output and transfer characteristics of the TFTs with different annealing temperatures. With an increase in the ZTO annealing temperature from 320 to 540 °C, the augmentative saturation region I_DS_ can be detected, as shown in Figure 6a. All electrical parameters of ZnSnO/ZrO_2_ TFTs containing the increased μ_sat_ and the I_on/off_, as well as the decreased V_TH_ and the reduced SS, can be seen in Table 1, which proves that the annealing treatment is an effective method to eliminate hydroxyl-related defects and improve the electrical properties of TFTs. According to our previous research, there are numerous grain boundaries (GB) in the polycrystalline inverse rutile crystal structure Zn_2_SnO_4_, as shown in Figure 5b [8,24]. It is generally acknowledged that grain boundaries exhibit lower electrical conductivity than within the grains, which lies in the strong electron scattering by the generated defects resulting from the abrupt change in atomic arrangement (lattice distortions) and the discontinuity in chemical bonding (unsaturated dangling bonds) at the grain boundaries. Compared with the low-quality grain boundaries obtained by low-temperature annealing, the grain boundary dangling bond-related defects in the high-quality grain boundaries can be passivated by high-temperature annealing, and improved electrical performance can be noticed. Yao et al. conducted experiments to verify that there was an intrinsic electric field near the grain boundaries, which could drive electrons to accumulate at the high-quality grain boundaries [25]. Chen et al. also discovered that the high-quality grain boundaries exhibit a significantly higher electrical conductivity than that within the grains [26]. However, the grain boundaries will spontaneously attract oxygen vacancies to diffuse and concentrate due to lattice distortion. When the ZnSnO films experience rapid cooling after high-temperature annealing, a large number of oversaturated oxygen vacancies defects are unable to combine with the oxygen in the air and remain at the grain boundaries to form continuous localized deep trap states, which can directly capture free electrons in the conduction band, as shown in Figure 5b. The trapped electrons cannot participate in conduction, and a negative space charge layer will form at the grain boundary meanwhile, which will raise the grain boundary barrier and further hinder the carriers from transporting across the grain boundaries, ultimately reducing the overall carrier mobility.

When the TFTs are conserved in a dark vacuum environment for 10 days, all devices exhibit obvious elevated electrical properties, as shown in Figure 6c,d. As demonstrated in Table 1, the 430 °C-annealed TFT after 10-day vacuum aging exhibits better electrical properties in comparison to 540 °C-annealed samples without experiencing aging, which implies that the aging treatment is an effective method to reduce the annealing temperature. Taking the 540 °C-annealed samples under the vacuum aging condition for systematic study, Figure 6c demonstrates a higher I_DS_ in the saturation region, and Figure 6d reveals significantly improved electrical parameters, including the markedly increased I_on/off_ from 2.0 × 10^4^ to 1.3 × 10^5^ and the boosted μ_sat_ from 2.2 to 5.8 cm^2^·V^−1^·s^−1^. The decreasing V_TH_ and SS can also be noticed in Table 1, which can be explained by the relaxation of various defects. Firstly, the dangling bonds defects at the grain boundaries gradually underwent reconfiguration and combined to saturate after 10-day aging; hence, the reducing defect density (correlated with SS) can be noticed. In addition, the aforementioned quick annealing treatment caused the oxygen vacancy defects concentration to stay in a high-energy non-equilibrium state. In order to reduce the overall free energy and achieve relaxation, oxygen vacancies need spontaneous rearrangement through combination with lattice interstitial oxygen, as shown in the Figure 5c. Low-concentration uniformly distributed oxygen vacancies can play a role in providing carriers. Therefore, reduced energy leads to the quick transmission of liberating electronics along path I. From the perspective of charge balance, the Sn element in ZnSnO oxide results in variable valence states of +2/+4. Excessive oxygen vacancies located at the grain boundaries may lead to local charge imbalance. In order to maintain overall electrical neutrality, the oxygen vacancies need to spontaneously relax. A portion of oxygen vacancies transfer charge with adjacent cations (such as reducing Sn^4+^ to Sn^2+^) to provide additional free electrons, and another part adjusts the defects charge distribution by undergoing rearrangement with the lattice atoms. Therefore, the local charge balance and the reducing defects system is obtained, which implies that significantly improved μ_sat_ is available. Finally, large residual internal stress is introduced during the ultraviolet curing and annealing process due to the mismatch in thermal expansion coefficients between the metal oxide film and silicon substrate. The relaxation of internal stress can be realized through the vacancy migration and arrangement at room temperature simultaneously. Accordingly, in terms of the vacuum aging process, not only does it reduce the trap density at the grain boundaries but it also improves the interface quality. Eventually, the sub-threshold swing and device mobility are both optimized [27].

When the ZTO samples are preserved in an air-ambient environment for 10 days, the evident promotion of electrical parameters can be seen, as shown in Table 1. Compared with 540 °C-annealed ZTO samples, the 10-day air-ambient aged TFT demonstrates a higher I_on/off_ up to 7.0 × 10^6^, larger μ_sat_ reaching up to 5.9 cm^2^·V^−1^·s^−1^, the lower V_TH_ of 0.44 V and smaller SS of 0.07. The extremely low SS is close to the reported limit value and implies that a clean interface is obtained [7]. Generally, the all-solution-based ZTO TFTs demonstrate a relatively low μ_sat_ of 0.57~2.9 cm^2^·V^−1^·s^−1^ despite experiencing separate aging treatment, as shown in Table 1. However, the 540 °C-annealed ZTO TFT with 10-day air-ambient aging surpassed the ever-achieved characteristics of TFTs, verifying that the combined post-annealing rapid cooling and aging treatment process can greatly boost the electrical performance. The exhibited superior electrical property can be attributed to two reasons: one reason is that the aforementioned liberating electrons realize quick transmission along transmission path I due to defects relaxation, and the adsorbed oxygen molecules from the environment can illuminate oversaturated oxygen vacancies defects and passivate the dangling bonds; another crucial reason is that the H_2_O molecular in the air may play the electron donors role, and the new introduced electrons achieve rapid transmission along transmission path II, as illustrated in Figure 5d. The H_2_O molecular diffusion in an air environment can be written as the following reaction:

(4) $$H_{2}O\;⇌\;H_{2}O^{+}+\;e^{-}$$

which implies that the H_2_O molecular effect is a more important role of electronic donors and can contribute to the mass of electrons in the channel. Consequently, the TFTs aging in the ambient air demonstrate more excellent electrical properties in comparison to the aforementioned vacuum environment aging samples. Huang et al. reported that the doping of 1.67 mol deionized water in 2ME ZTO solution clearly raised the μ_sat_ from 0.92 to 2.11 cm^2^·V^−1^·s^−1^ [28]. However, we found that adding deionized water is liable to disrupt the original organic solvent balance, causing the precursor to precipitate and form hydroxide colloids. The aging diffusion method, by contrast, is characterized by its simplicity and demonstrates more promising applications in terms of performance improvement. It is worth noting that the aging-enhanced approach can be easily extended to other types of TFTs based on different metal oxides as gate dielectrics and different semiconducting channels in our previous studies [8]. The air aging-treated ZnSnO/DyO_x_ TFTs demonstrate an increased μ_sat_ of 2.5 from 0.57 cm^2^·V^−1^·s^−1^ and increased I_on/off_ of 2.0 × 10^6^ from 7.8 × 10^4^, as demonstrated in Table 1. The air aging-processed ZnSnO/Yb_2_O_3_TFTs display an elevated μ_sat_ of 5.9 from 0.556 cm^2^·V^−1^·s^−1^ and higher I_on/off_ of 8.7 × 10^6^ from 1.4 × 10^5^ [6]. The air aging-handled InZnO/DyO_x_ TFTs also exhibit an enhanced μ_sat_ of 12.6 from 11.8 cm^2^·V^−1^·s^−1^ and incremental I_on/off_ of 1.0 × 10^9^ from 4.66 × 10^7^ [7]. All of the above results verify that the facile air aging method improves the electrical properties of all-solution-based TFTs. Similarly, Bae et al. pointed out that sol–gel IGZO TFTs demonstrate a higher I_on/off_ during the aging diffusion stage [29].

When the 540 °C-annealed TFT is preserved in a vacuum environment for 20 days, although the slightly decreased μ_sat_ from 5.8 to 5.0 cm^2^·V^−1^·s^−1^ can be monitored, the significantly improved I_on/off_ from 1.3 × 10^5^ to 9.3 × 10^6^ as well as the continuously declining V_TH_ can be detected according to the initial transfer characteristic curve, as demonstrated in the inset of Figure 6e. It is worth noting that the small off-state current is lower than the testing instrument’s current detection lower limit, which indicates the device channel is completely depleted and there is no detectable leakage current. In other words, the V_GS_ can effectively control the leakage current resulting from oxygen vacancies relaxation. In addition, the decreasing SS and the clean interface imply that the stable vacuum environment prevents the contamination of air impurities, which benefits the electron transport. When the corresponding MOS devices are fabricated in an air environment, a large number of water molecules will diffuse into the dielectric film, resulting in an increase in measured capacitance. Even if the TFT devices are aged under different conditions, a dynamic equilibrium will be established and will not change the calculated C_OX_, as shown in Figure S1. To avoid overestimating the μ_sat_, we adopt the upper-limit value of the intrinsic capacitance of the gate insulating layer. It is worth noting that the 30-day vacuum aging TFT did not exhibit the typical output and transfer characteristics resulting from stress-induced device breakdown failure after bias illumination stability experiments, which can be verified by the approach 0 capacitance value, as shown in Figure S1.

When the 540 °C-annealed sample is preserved in an air atmosphere for 20 days and 30 days, an increasing number of trapped electrons are liberated, and the left deviation transfer characteristic can be detected, as shown in Figure 6d. When the retracing voltage affects the aging device, a part of the inducing electrons will fill the localized states, and in the other part, free electrons can transmit quickly in the channel layer and raise the I_DS_. Therefore, decreasing hysteresis can be noticed. Unlike the continuously decreasing I_off_ with continuously increasing I_on/off_ of 9.3 × 10^6^ exhibited in vacuum-aged devices, higher I_off_ as well as significantly reduced I_on/off_ of 2.4 × 10^5^ signify that air aging TFTs show poor leakage performance, as demonstrated in Figures S2 and S3. Meanwhile, the mass of water molecules diffused in the interface between the active layer and dielectric layer, which may generate new metamorphic hydroxyl defects and obstruct the transport of electrons according to the following reactions:

(5) $$M+H_{2}O+O_{2}\;⇌\;M(\mathrm{OH}{)}_{X}$$

where M represents Zn, Sn and Zr. Consequently, the deteriorated electrical performance featured with the decreasing μ_sat_ and increasing SS can be observed as displayed in Figure S3 and Table 1. Consequently, the TFTs aging in vacuum conditions demonstrate wider aging time windows in comparison to devices in the air aging condition for 20 days, as demonstrated in Figure S3. Long et al. reported that replacing the top part of ZrO_2_ with an Al_2_O_3_ composite material layer can significantly enhance the reliability of the ultrathin capacitor [20].

**Figure 7:**
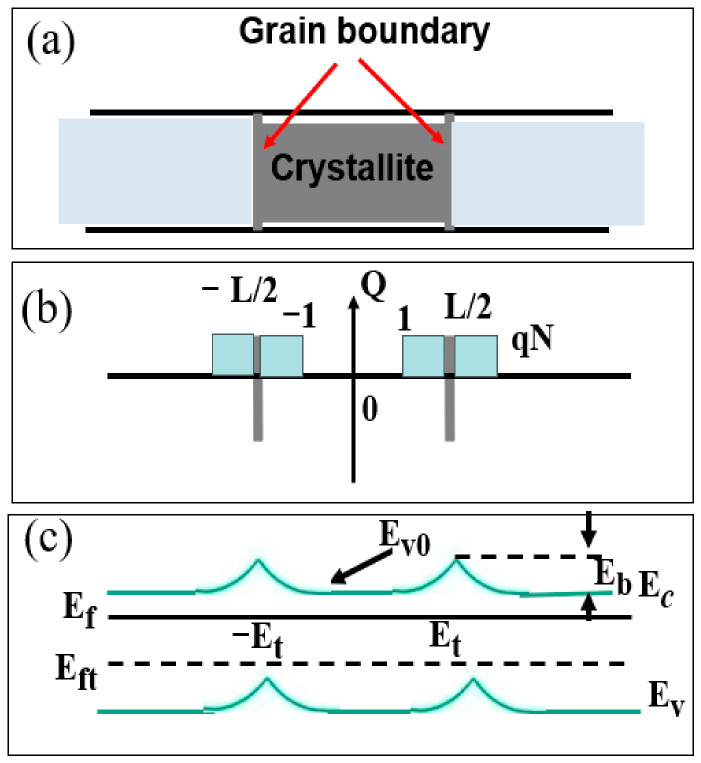
(**a**) Typical diagram of polycrystalline materials. (**b**) The charge trapping model at grain boundaries. (**c**) The formation and influence of grain boundary barriers.

Seto proposed a classic polycrystalline material carrier transmit model and grain boundary barrier height calculation formula based on energy band theory [30]. Figure 7a displays a typical diagram of polycrystalline materials. There are a large number of dangling bonds, vacancies and impurity atoms located at the grain boundaries, which can introduce localized deep/shallow trap energy levels in the semiconductor band gap and capture the free carriers (such as electrons). As demonstrated in Figure 7b, when the negatively charged electrons (represented by “qN”) are trapped in the grain boundary, the positive space charge regions (depletion layers) will be induced in the grains on both sides of the grain boundary to maintain electrical neutrality and form a structure similar to a “double layer”. An intrinsic electric field within the grains can be generated by the negative charges located at the grain boundaries, which result in the pined Fermi level and the bend energy bands, as illustrated in Figure 7c. Both the conduction band bottom (E_c_) and the valence band top (E_v_) bulge upward at the grain boundaries; the resultant potential barrier formation (E_b_) hinders electrons from transmitting from one grain to another, resulting in an increase in resistivity and a decrease in μ_sat_ [30]. In addition to this, the barriers at the junction of the ZTO semiconductor (high work function of 5.3 eV) and aluminum electrode (low work function of 4.06 eV) will prevent electron transmission. Only the abundant electrons climb these barriers, if they can transfer in the channel layer. Matare et al. proposed that the carrier transport across the grain boundary is mainly governed by the thermionic emission mechanism and the tunneling mechanism due to the existence of grain boundary barriers, and, thus, the effective mobility of the device is closely related to the barrier height and temperature. [31,32]. Abundant traps in a low-quality grain boundary will capture charge carriers and form a space charge region, resulting in band bending, and the forming E_b_ can reach 0.3–0.6 eV. High-temperature annealing can reduce E_b_ to 0.1 eV and benefit electron transport [33]. According to the previous analysis, more and more stimulated carriers can overcome the E_b_ and transmit in the conduction band as the aging days are prolonged.

**Figure 8:**
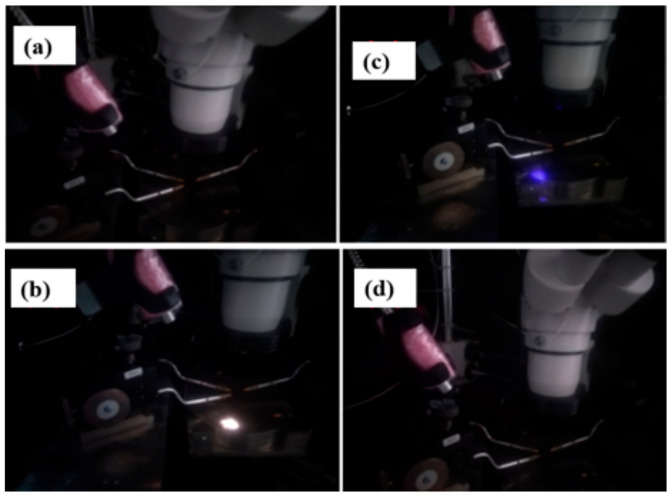
The experiments of the PBS under (**a**) darkness and the PBIS under (**b**) white LEDs, (**c**) blue light and (**d**) ultraviolet radiation conditions, respectively.

**Figure 9:**
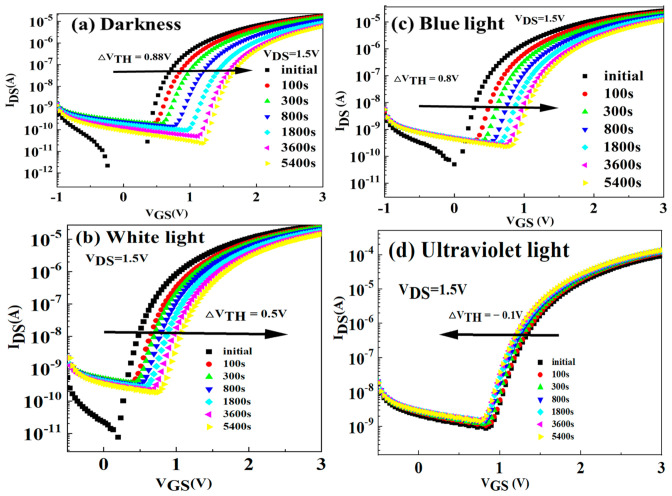
The PBS results under (**a**) darkness and the PBIS results under (**b**) white LEDs, (**c**) blue light and (**d**) ultraviolet radiation conditions, respectively.

**Figure 10:**
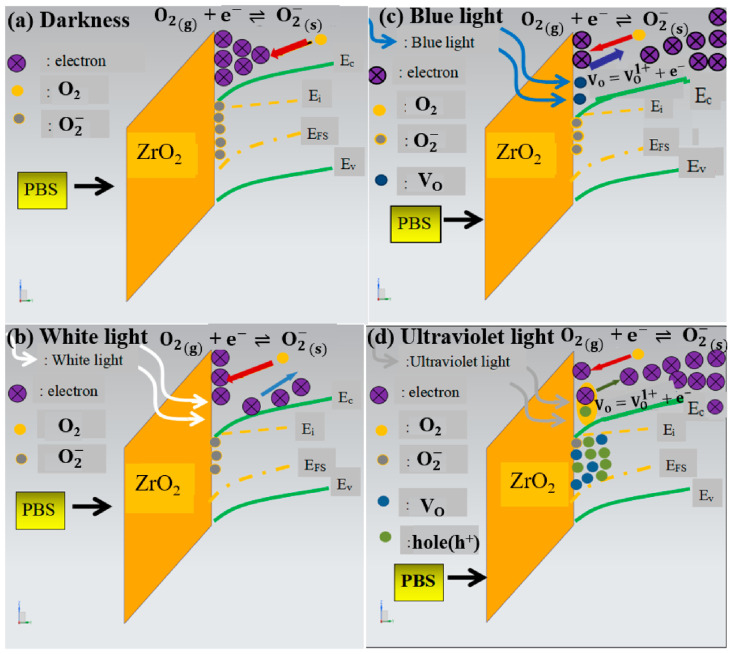
The energy band diagrams of the PBS under (**a**) darkness and the PBIS under (**b**) white LEDs, (**c**) blue light and (**d**) ultraviolet radiation conditions, respectively.

The positive bias stability (PBS) and positive bias illumination stability (PBIS) are essential for their commercial utilization in active-matrix liquid crystal displays and organic light-emitting diodes. The PBS experiment was engaged on 540 °C vacuum-annealed ZTO samples under a dark environment, as demonstrated in Figure 8a. The PBIS experiments were conducted under various illumination conditions, including white light, blue light and ultraviolet radiation, with constant 0.1 mw light intensity, as shown in Figure 8b to Figure 8d. The different transfer characteristics under PBS and PBIS experiments are illustrated in Figure 9. Figure 10 displays a trapping and detrapping model on the illuminated interface. The source and drain electrodes remained grounded, while the gate bias voltage of 1.5 V was stressed on the ZTO/ZrO_2_ TFT for 5400 s. The threshold voltage shift (ΔV_TH_) is mostly used to evaluate the TFT bias stability on the basis of the following expression:

(6) $${\Delta V}_{\mathrm{TH}}=V_{\mathrm{TH}}(t)-V_{\mathrm{TH}}(0)$$

where V_TH_(t) and V_TH_(0) denote the measured time V_TH_ value and initial value on the basis of the transferring curve.

The obvious ΔV_TH_ of 0.88 V can be noticed, as demonstrated in Figure 9a. The solution-prepared film forms a porous structure during the spinning and annealing process; the O_2_ molecules in the ambient air easily pass through the channel layer and gather in the interface, as shown in Figure 10a. As a result, a mass of transporting electrons in the channel have been depleted due to the following reaction:

(7) $${O_{2}}_{\left(g\right)}\;+\;e^{-}⇌\;{O_{2}^{-}}_{\left(s\right)}$$

where O_2(g)_ and O_2_^−^_(s)_, respectively, represent the neutral and charged oxygen molecules; e^−^ denotes electrons [6]. A stronger electric field with a larger turn-on voltage is needed to induce the electrons to transmit in the conduction band. Consequently, the obvious enlarging Δ*V*_TH_ of 0.88 V was subsequently noticed. The energy band diagram also demonstrates the positions of Ec (conduction band edge), Ev (valence band edge), Ei (intrinsic Fermi level), EFS (Fermi level pinning). After 5400 s PBS, the TFT demonstrates an increased SS from 0.07 to 0.11 and suggests the generated amounts of O_2_^−^ defects in the interface. The charge trapping model is commonly used to explore the relationship between stress time (t) and V_TH_, which generally coincides with the following stretched exponential model:

(8) $$\Delta V_{\mathrm{TH}}=\Delta {V_{\mathrm{TH}}}_{0}\left[1-e^{-{\left(\frac{t}{\tau }\right)}^{\beta }}\right]$$

where ΔV_TH_(t) and ΔV_TH_(0) denote the measured time ΔV_TH_ value and initial value.

V_TH0_ is the VTH at infinite time, *β* is on behalf of the stretched exponent, *τ* represents the characteristic detrapping time [23]. The relationship between Δ*V*_TH_ and time (*t*) matched well with the exponential model, as demonstrated in Figure S4, indicating that the carrier trapping assumption is the conclusive mechanism model for adding Δ*V*_TH_.

The white light can be acquired using a standard Aurora 4000 LED lamp source, as demonstrated in Figure S5a. The outside of the PBIS testing equipment has a black cover, which is used to eliminate the influence of ambient light on the PBIS. A clear PBIS testing equipment picture is added in Figure S5b. The decreasing Δ*V*_TH_ of 0.5 V is available under the white light PBIS, as shown in Figure 9b, which is slightly smaller than the recent report (0.56 V) [18]. The sub-band-gap energy of white light can activate the above-mentioned captured electrons and stimulate them to the conduction band, as illustrated in Figure 10b; thus, lower Δ*V*_TH_ is accessible. The parallel *V*_TH_ shift and the constant SS of 0.09 imply that no new defects have been generated in the interface.

The obvious Δ*V*_TH_ of 0.8 V is observable when the PBIS experiment is engaged by the blue light illuminating, as shown in Figure 9c. The electron–hole pair model is often used to investigate the illumination instability phenomenon on the basis of the following photon energy formula

(9) $$E=\frac{hc}{\lambda }$$

where E, h, c and λ represent the photon energy, Planck constant of 4.13567 × 10^−15^ eV·s, light velocity of 3 × 10^8^ m s^−1^ and wavelength, respectively. The λ of blue light is 450 nm, and the calculated E is 2.79 eV [6]. When the photon energy surpasses the ionization energy of oxygen vacancy, the oxygen vacancy in the channel layer will ionize and activate plenty of electrons, according to the following reaction.

(10) $$\;V_{O}=V_{O}^{1+}+e^{-}$$

(11) $$V_{O}=V_{O}^{2+}+2e^{-}$$

The ionization energies of these reactions are 2.0 eV and 2.3 eV, respectively [7]. Therefore, the blue light with a photon energy of 2.79 eV can motivate abundant electrons to the conduction band, and a high I_on/off_ of 2.62 × 10^6^ is available. Meanwhile, the newly generated defects containing V_O_^1+^ and V_O_^2+^ may stay in the bulk of the channel layer or the interface. For the sake of simplicity, only the V_O_^1+^ is marked in Figure 10c. These interfacial defects will impede the electronic transport and increase the device instability, which results in a remarkable increase in SS from 0.082 to 0.173.

Under the illumination of 300 nm ultraviolet light, the distinct left deviation Δ*V*_TH_ of −0.1 V can be noticed, as displayed in Figure 9d. The device demonstrates an increased I_off_ and worse leakage property after PBIS experiments, which results from the generation of various interface defects. On the basis of Equation (9), the calculated photon energy of ultraviolet light can reach up to 4.18 eV, which exceeds the energy gap of the ZTO film (3.6 eV) [6]. As a result, a large number of electron–hole pairs are excited into the conduction band, as illustrated in Figure 10d, and the I_on_ is increased to 1.44 × 10^−4^ A. As the bias time is prolonged, an increasing number of photogenic charge carriers are inspired and compensate for the aforementioned trapping effect. As a result, a smaller threshold voltage was noticed; thus, the transmitting curve shifted to the left, and a distinctive negative Δ*V*_TH_ of −0.1 V is available. Except for the mass of forming V_O_^1+^ and V_O_^2+^ defects, it is worth mentioning that the generating abundant hole (h^+^) obviously degraded the device’s electrical property, and the remarkably increased SS of 0.16 implies the unstable interface is available.

Among various PBIS results, the white light-illuminated TFT shows the most stable electrical properties, characterized by the lowest positive Δ*V*_TH_ of 0.5 V and a constant SS of 0.09. All the activated carriers, simulated electron–hole pairs and ionized oxygen vacancies can impact the interface stability and generate a great variety of Δ*V*_TH_. On the contrary, the distinctive Δ*V*_TH_ can also be used for detecting diverse illuminating lights and demonstrate the potential applications in photoelectric detectors. In addition, the carrier concentration in the photocatalytic process can be in situ regulated through using the field-effect regulation method of TFT, which provides a new research platform for studying the dynamics of photocatalytic charges in the future [34,35,36,37,38].

## 4. Conclusions

In this work, the rapid annealing cooling with aging treatment process was innovatively proposed to build high-performance thin-film transistors. Rapid annealing cooling brings a large amount of supersaturated oxygen vacancies and hinders electron transport in high-temperature annealing. After a certain humidity and time aging process, the relaxation of various defects as well as water molecules, the electron doner role increases the electrical performance. In comparison with air aging, vacuum aging shows a lower leakage current and longer aging day window in regulating the oxygen vacancies concentration. In addition, the distinctive illumination interface stability demonstrates potential applications in photoelectric detectors and ultimately realizes “photovoltaic detection—catalytic drive”-integrated devices.

## Figures and Tables

**Table 1:** Electrical parameters of ZnSnO/ZrO_2_ TFTs with various annealing temperatures and aging conditions.

ZnSnO Anneal Temperature	Aging Condition	Aging Days	μ_sat_ [cm^2^ V^−1^ S^−1^]	I_on_/I_off_	V_TH_ [V]	SS [V dec^−1^]
320 °C	\	\	\	2.7 × 10^2^	1.35	0.32
430 °C	\	\	1.3	2.0 × 10^3^	0.76	0.18
540 °C	\	\	2.2	2.0 × 10^4^	0.6	0.16
320 °C	vacuum aging	10 days	0.16	1.54 × 10^3^	0.95	0.29
430 °C	vacuum aging	10 days	2.68	2.6 × 10^4^	0.7	0.12
540 °C	vacuum aging	10 days	5.8	1.3 × 10^5^	0.54	0.11
540 °C	vacuum aging	20 days	5.0	9.3 × 10^6^	0.47	0.09
540 °C	air aging	10 days	5.9	7 × 10^6^	0.44	0.07
540 °C	air aging	20 days	3.6	2.4 × 10^5^	0.38	0.1
540 °C	air aging	30 days	3.2	1.1 × 10^4^	0.37	0.21
ALD- ZnSnO/ZrO_2_ [18]	\	\	8.5	~10^6^	1.3 (V_GS_ = 10 V)	\
500 °C ZnSnO/ZrO_2_ [16]	\	\	2.5	10^6^	1	0.23
560 °C ZnSnO/ZrGdOx [9]	\	\	2.9	1.1 × 10^6^	0.7	0.092
500 °C ZnSnO/DyOx [8]	\	\	0.57	7.8 × 10^4^	0.10	0.10
500 °C ZnSnO/DyOx [8]	air aging	10 days	2.5	2.4 × 10^6^	0.5	0.08
600 °C In_2_O_3_/SrO_x_ [11]	\	\	5.61	10^7^	1.23	0.11
500 °C In_2_O_3_/Yb_2_O_3_ [12]	\	\	4.98	10^6^	0.38	0.07
300 °C In_2_O_3_/ZrO_2_ [23]	\	\	4.42	10^7^	0.31	0.078

## Data Availability

The data are available from the corresponding authors upon reasonable request.

## Supplementary Materials

The following supporting information can be downloaded at: https://www.mdpi.com/article/10.3390/nano16140861/s1, Figure S1: Areal capacitance of the 560 °C-annealed ZrO_2_ thin film with different aging days (a) under the vacuum condition, (b) under the air condition. Figure S2: Output characteristics of the 540 °C ZnSnO/560 °C ZrO_2_ TFT under the condition of air aging for (a) 10 days, (b) 20 days and (c) 30 days. (d) Transfer characteristics comparison of the 540 °C ZnSnO/560 °C ZrO_2_ TFT under air environment within the aging days ranging from 0 to 30 days. Figure S3: The μ_sat_ and I_on/off_ comparison of 540 °C ZnSnO/560 °C ZrO_2_ TFT under vacuum aging and air aging environment. Figure S4: The Δ*V*_TH_ and stress time (s) matched well with the exponential model. Figure S5a: The spectrum of a standard Aurora 4000 lamp source used for illumination. Figure S5b: The clear PBIS testing equipment picture.
