# Supplementary material for: Aging-Enhanced High-Performance Zinc Tin Oxide Transistors and Exploration in Illumination Interface Stability
Source: Nanomaterials (Basel). 2026 Jul 13;16(14):861. doi: 10.3390/nano16140861 (PMC13414651; doi:10.3390/nano16140861)
Supplement: Supplementary file 1 [file nanomaterials-16-00861-s001.zip › nanomaterials-4310351-supplementary.pdf]

Supporting Information

Aging-Enhanced High-Performance Zinc Tin Oxide Transistors and Exploration in Illumination Interface Stability

Bing Yang [1,*,†], Qiao Guo [2,†], Hongmin Li [3], Gang He [4,*], Shanshan Jiang [4], Longwei He [5], Xiang Li [1,*] and Peng Yu [1]

[1] School of Intelligent Manufacturing, Anhui University of Applied Technical, Hefei 230011, China
[2] School of Computer and Information Technology, Anhui University of Applied Technical, Hefei 230011, China; guoqiao@uta.edu.cn
[3] Hefei BOE Optoelectronics Technology Co., Ltd., Hefei 230011, China
[4] Radiation Detection Materials & Devices Lab, Anhui University, Hefei 230601, China
[5] Pioneer Film Materials (Anhui) Co., Ltd., Hefei 230011, China
* Correspondence: yangb@uta.edu.cn (B.Y.); hegang@ahu.edu.cn (G.H.); lixiang20@uta.edu.cn (X.L.)
† These authors contributed equally to this work.

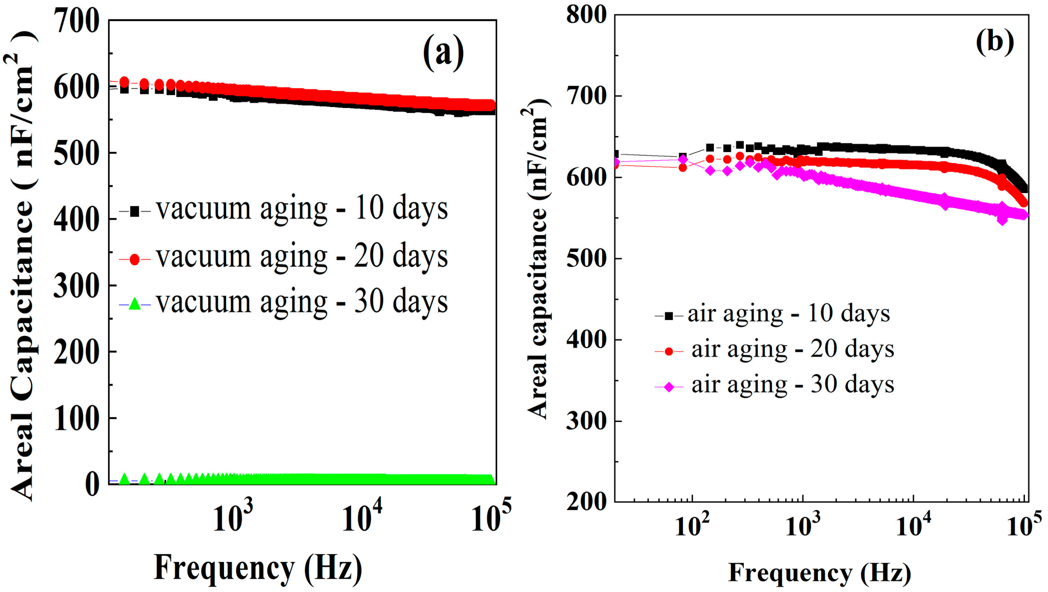

Fig.S1: Areal capacitance of the 560 °C-annealed ZrO_2 thin film with different aging days (a) under the vacuum condition. (b) under the air condition.

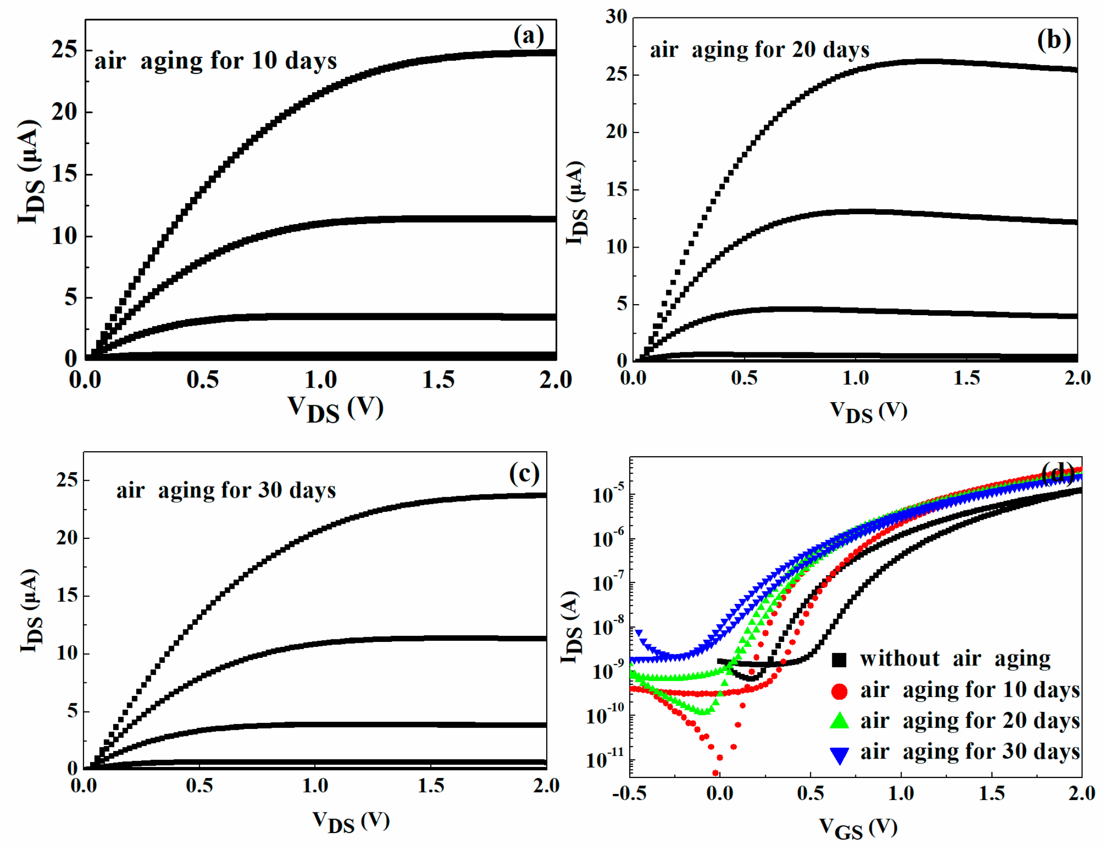

Fig.S2. Output characteristics of the 540 ºC ZnSnO/560 ºC ZrO_2 TFT under the condition of air aging for (a)10 days, (b) 20 days and (c)30 days. (d) Transfer characteristics comparison of the 540 ºC ZnSnO/560 ºC ZrO_2 TFT under air environment within the aging days range from 0 to 30 days

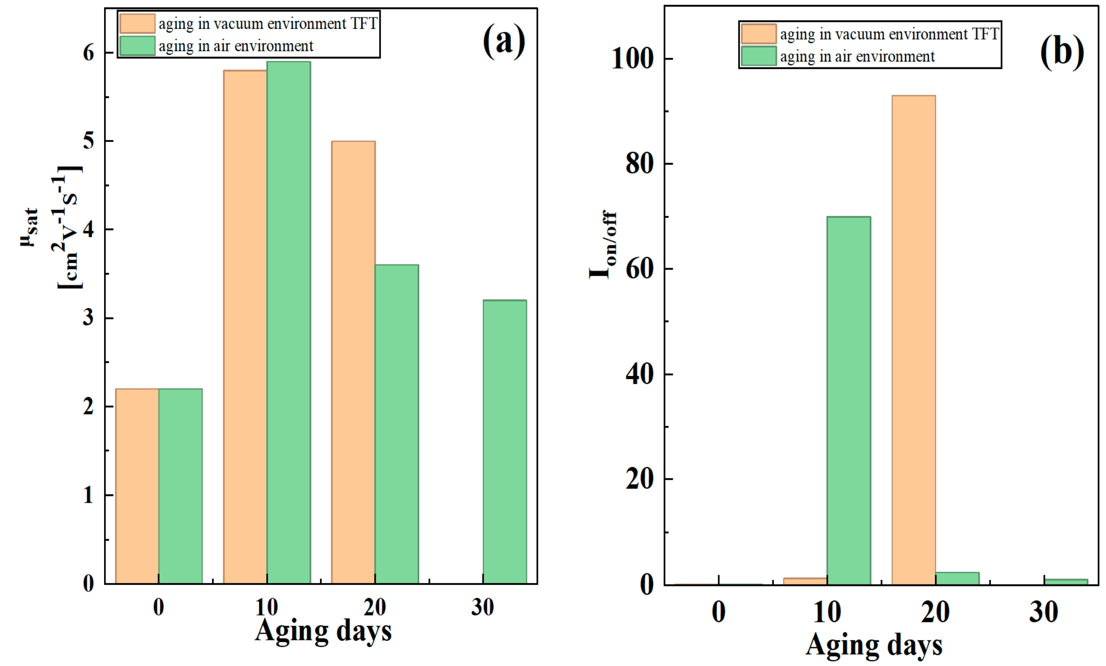

Fig.S3: The μ_{sat} and $I_{on/off}$ comparison of 540 ºC ZnSnO/560 ºC ZrO_2 TFT under vacuum aging and air aging environment

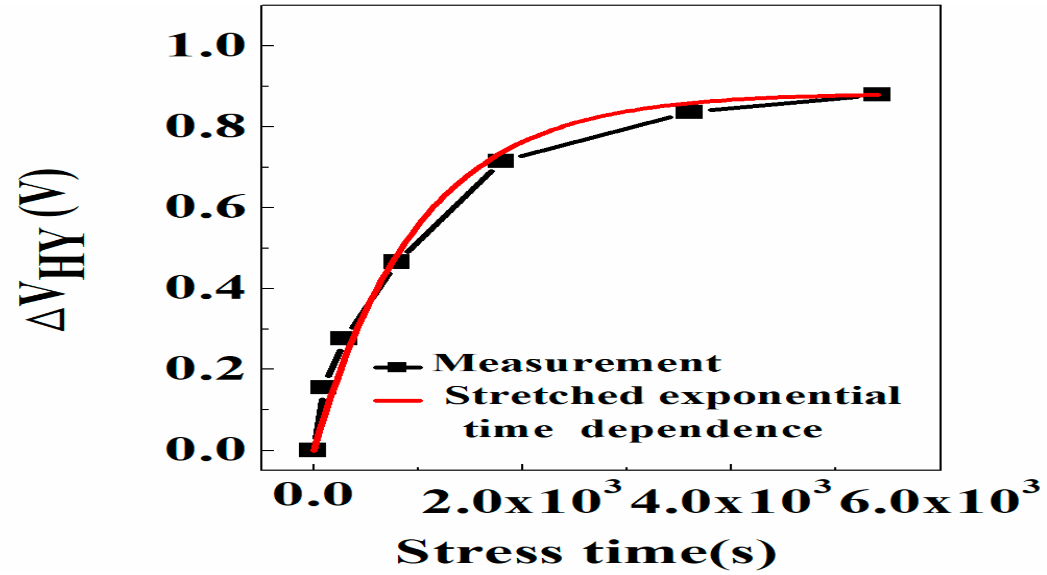

Fig.S4: The ΔV_{TH} and stress time(*s*) have matched well with the exponential model.

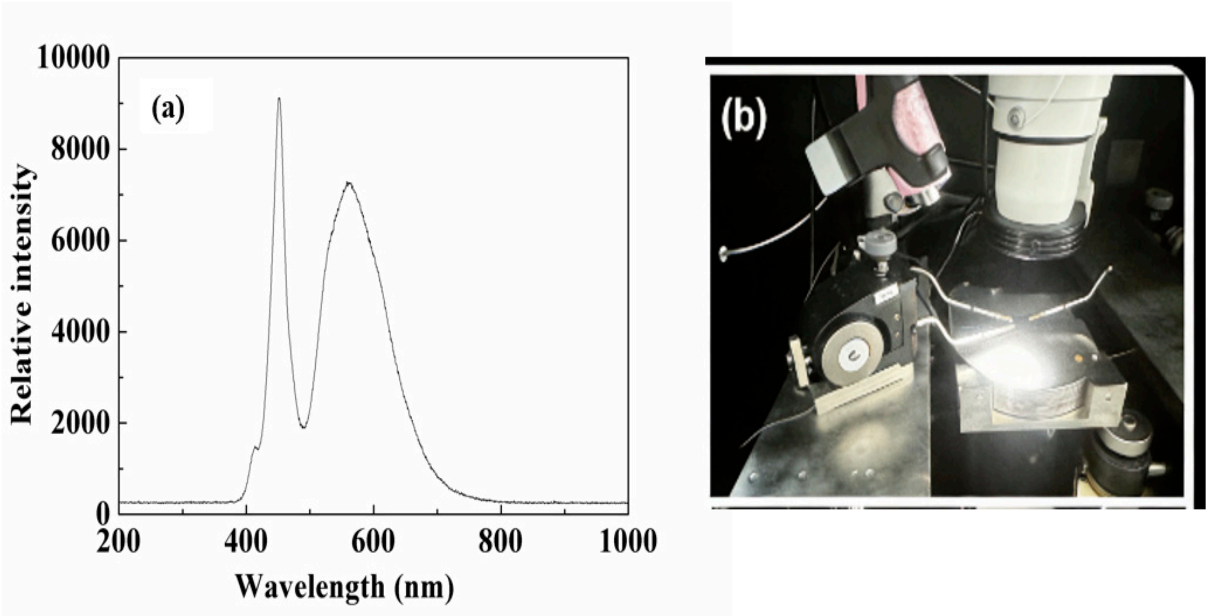

Figure S5(a): The Spectrum of a standard Aurora 4000 lamp source used for illumination. **Figure S5(b)**: The clear PBIS testing equipment picture.
